# Efficacy analysis of serum ferritin, Hcy, CRP combined with fat ultrasound parameters in predicting metabolic dysfunction-associated steatotic liver disease

**DOI:** 10.3389/fmed.2025.1637694

**Published:** 2025-12-17

**Authors:** Haitao Yu, Liju Ma, Yuxiang Chen, Juan Liu, Linjian Li, Zhifang Yang, Jing Xu, Bei Guo

**Affiliations:** 1 Department of Ultrasound Medicine, The First Hospital of Zhangjiakou, Zhangjiakou, China; 2 Department of Health Management Section, The First Hospital of Zhangjiakou, Zhangjiakou, China; 3 Department of Ultrasonic Diagnosis, The Hospital of 81 ST Group Army PLA, Zhangjiakou, China; 4 Department of Chinese Medicine, The First Hospital of Zhangjiakou, Zhangjiakou, China; 5 Department of Nuclear Medicine, The First Hospital of Zhangjiakou, Zhangjiakou, China

**Keywords:** metabolic dysfunction-associated steatotic liver disease, serum ferritin, homocysteine, C-reactive protein, fat ultrasound parameters

## Abstract

**Objective:**

To investigate the efficacy of combined prediction of serum ferritin, Hcy, CRP, and fat ultrasound parameters in metabolic dysfunction-associated steatotic liver disease (MASLD).

**Methods:**

One hundred and two MASLD patients admitted to our hospital from November, 2021 to November, 2023 were selected as the MASLD group, and 106 healthy subjects were selected as healthy group on the basis of the principle of 1:1, and the healthy group was not accompanied by any basic diseases. The differences in demographic data, medical records and biochemical indicators between the MASLD group and the healthy group were analyzed by single factor analysis. The suspected influencing factors of MASLD were screened by single factor analysis. Apply multivariate Logistic regression to examine the independent predictors of MASLD. Employ ROC curve to anticipate the effect of single and combined indicators.

**Results:**

Single factor analysis revealed that the SF, Hcy, CRP, and CAP level in MASLD group were significantly higher than healthy control group (*p* < 0.05). Multivariate Logistic analysis displayed that SF, Hcy, CRP, and CAP were independent predictors of alcoholic fatty liver disease (*p* < 0.05). ROC curve displayed that the area under the curve of the joint prediction of SF, Hcy, CRP, and CAP was 0.941, which was significantly higher than the area of SF, Hcy, CRP, and CAP alone.

**Conclusion:**

The combined application of serum SF, Hcy, CRP, and CAP has a high predictive efficiency for MASLD, which can be employed as an important reference indicator for early clinical diagnosis and intervention.

## 1 Introduction

Metabolic dysfunction-associated steatotic liver disease (MASLD) is a clinicopathological syndrome featured by excessive fat deposition in hepatocytes. The diagnosis of MASLD requires the exclusion of excessive alcohol consumption and other definite liver injury factors (1). The incidence of MASLD is increasing year by year globally, and it has become one of the major types of chronic liver disease, which seriously affects public health. Its pathogenesis is complex, involving insulin resistance, inflammatory response, oxidative stress, lipid metabolism disorders and other aspects (2, 3). With the increasing incidence of metabolic diseases such as type 2 diabetes, obesity, and metabolic syndrome, the prevalence of MASLD has significantly increased, imposing a heavy burden on the social medical system. Early diagnosis and effective intervention are essential for the management of MASLD. However, the current diagnosis of MASLD mainly relies on imaging examination and serological markers, and the predictive efficacy of single indicators is limited, and there is a high rate of missed diagnosis and misdiagnosis (4). Therefore, finding more sensitive and specific predictors has become a current research hotspot.

Serum ferritin (SF) is the main form of iron storage in the human body, and its abnormal increase is closely related to oxidative stress and inflammatory response in patients with MASLD, which may participate in occurrence and development of MASLD by promoting lipid peroxidation and inflammatory response (5). Homocysteine (Hcy) is an intermediate product of methionine metabolism, and its elevated level reflects the impairment of liver metabolic function and is closely related to the progression of MASLD (6). C-reactive protein (CRP), as an acute phase protein, is a sensitive index to evaluate the systemic inflammatory response, and its increase in MASLD patients may indicate the presence of liver inflammation (7). In addition, fat ultrasound parameters such as controlled attenuation parameter (CAP) can quantitatively valuate the degree of liver fat change, which provides an intuitive basis for the diagnosis of MASLD. Therefore, this study aimed to explore the predictive efficacy of the combined application of these indicators for MASLD, to provide a scientific basis for the clinical diagnosis of MASLD.

## 2 Data and methods

### 2.1 Basic data

One hundred and two MASLD patients of our hospital from November 2021 to November 2023 were selected as MASLD group, and 106 healthy subjects were picked as healthy group according to the principle of 1:1, and healthy group was not accompanied by any underlying diseases. This study got approval of the hospital ethics committee, all patients and their families voluntarily signed informed consent.

### 2.2 Inclusion criteria and exclusion criteria

*Inclusion criteria*: (1) referring to the diagnostic criteria of MASLD in the “Non-alcoholic fatty liver disease: diagnosis and investigation” (8), and ultrasound diagnosis met any two or more of the following: ① the overall liver showed a generally enhanced echo phenomenon; ② The internal hepatic duct structure was blurred and difficult to identify; ③ The ultrasonic signal in the far-field region of the liver was gradually weakened during the propagation process, and the echo attenuation phenomenon appeared. To obtain a more comprehensive and accurate assessment of liver steatosis, this study also utilized FibroScan and magnetic resonance imaging-estimated liver proton density fat fraction (MRI-PDFF) as auxiliary diagnostic tools to verify the results of the ultrasound examination, in order to make up for the limited sensitivity of ultrasound in detecting mild steatosis; (2) no secondary fatty liver disease such as total parenteral nutrition, hypothyroidism and anterior pituitary dysfunction; (3) those who were willing to cooperate with the study and accept ultrasound examination in our hospital; (4) patients with complete basic clinical data.

Exclusion criteria: (1) accompanied by specific diseases such as viral hepatitis, cirrhosis, hepatolenticular degeneration, autoimmune liver disease and drug-induced liver disease; (2) a history of alcohol abuse (>30 g/d for men and >20 g/d for women); (3) blood system and other diseases affecting serum SF, Hcy and CRP; (4) history of gastrointestinal surgery before the investigation; (5) with malignant tumor and abnormal cardiopulmonary function; (6) with cognitive and mental disorders; (7) patients with diabetes, obesity (BMI ≥ 30 kg/m^2^), or dyslipidemia (triglycerides ≥ 1.7 mmol/L or total cholesterol ≥ 5.2 mmol/L).

### 2.3 Sample size calculation

The sample size calculation was based on the level of CRP between MASLD patients and healthy individuals as reported previously (9). Assuming a significant level (*α*) of 0.05, a power (1−*β*) of 0.80, and 10% dropout, the total sample size was 101 participants in each group.

### 2.4 Methods

1. Basic data collection: The basic clinical data of all subjects were gathered through the electronic medical record system of our hospital, including age, gender, body mass index, history of basic diseases, liver function indicators, renal function indicators and other information.
2. All subjects were fasted for 8–10 h before the test, and in the morning, collected 6 mL of fasting elbow venous blood, then placed in the greenhouse for 10 min to wait for coagulation, and then centrifuged at 2,000 r/min for 10 min. The supernatant was stored in the refrigerator at −60 °C for testing. Chemiluminescence immunoassay (CLIA) was used to measure related laboratory indicators, including glucose and lipid metabolism indicators, SF, etc. Analyses were performed with the use of a fully automated chemiluminescence immunoassay analyzer (Roche, Roche Cobas E411). The serum Hcy and CRP levels were detected by ELISA. The human homocysteine enzyme-linked immunosorbent assay and human C-reactive protease immunoassay kits were purchased from R&D Systems China Co., Ltd.
3. Ultrasound examination: Use the FibroScan device (Echosens China, model 502) with the probe frequency of 1–8 MHZ. After 8 h of fasting, the subject was asked to lay supine with the right arm raised to ensure that the chest was fully exposed. Firstly, liver morphology, parenchymal echo characteristics, and far-field echo attenuation were preliminarily assessed by two-dimensional ultrasound. The right anterior lobe of the liver was selected as the detection area. The probe was placed vertically (with a depth of 10 cm) and positioned at the level of the liver capsule. The position of the sampling frame was adjusted to about 1–2 cm away from the liver capsule, and different sizes (7 × 9 cm and 3 × 4 cm) were set. The subject was asked to hold his breath for 1–3 s to capture a stable image. Controlled attenuation parameter (CAP) was measured by FibroScan® M (Echosens, Paris, France), and attenuation coefficient (ATT) was recorded, and each index was measured repeatedly three times and then averaged.

### 2.5 Observational index

(1) Univariate analysis of the differences in demographic data, medical records and biochemical indicators between the MASLD group and the healthy group; (2) Single factor screening for suspected influencing factors of MASLD; (3) Multivariate Logistic regression analysis was employed to analyze the MASLD independent predictors; (4) ROC curve was used to analyze the predictive efficacy of single and combined indicators.

### 2.6 Statistical treatment

SPSS 22.0 software was applied to analyze data. The Shapiro–Wilk test was used to determine whether the data followed a normal distribution. Measurements in accordance with normal distribution were represented as (*x̄* ± *s*). If comparing two sets of data, an independent sample *t*-test was used; if comparing three or more sets of data, a one-way analysis of variance (ANOVA) was employed. The independent sample *t*-test is applicable for comparing the means of two independent samples and can determine whether there is a significant difference between the overall means represented by these two sets of data; one-way ANOVA is used to analyze the influence of a categorical independent variable on a continuous dependent variable, by comparing the variances of different groups to determine whether there is a significant difference in the group means, thereby determining whether this categorical factor has a significant impact on the dependent variable. The count data of 2 groups were expressed as n (%), comparison between groups was analyzed by *χ*^2^ test. The *χ*^2^ test is mainly used to analyze the correlation between two or more categorical variables. By comparing the differences between the observed frequencies and the expected frequencies, it can determine whether there are significant differences in the distribution of the categorical variables among different groups. This test is applicable to the comparison of groups for count data in this study. The risk factors of MASLD were explored using logistic regression analysis. Logistic regression is a regression analysis method widely used in studies with binary dependent variables. It can analyze the relationships between multiple independent variables and the binary dependent variable by calculating the odds ratio (OR value) and its 95% confidence interval (CI), to assess the degree and direction of the influence of each independent variable on the dependent variable, thereby determining the potential risk factors of MASLD. When conducting the logistic regression analysis, the influence of potential confounding factors on the research results was considered. By including other variables that might affect MASLD in the logistic regression model for multivariate analysis, this method of multivariate adjustment can effectively control the interference of confounding factors, more accurately assess the true association between the target variable and MASLD, and improve the reliability and validity of the research results. The Receiver Operating Characteristic (ROC) curve was drawn to study the predictive value of combined detection of serum ferritin, homocysteine, C-reactive protein and fat ultrasound parameters for MASLD. The ROC curve is an important tool for evaluating the accuracy of binary diagnostic tests. It takes the false positive rate (1-specificity) as the abscissa and the true positive rate (sensitivity) as the ordinate. By plotting the sensitivity and specificity points at different critical values, a curve is formed. The area under the curve (AUC) is an important indicator for measuring the accuracy of the diagnostic test. The closer the AUC is to 1, the higher the accuracy of the diagnostic test. By comparing the ROC curves and AUC values of different indicators or their combined detection, the predictive ability and diagnostic value for MASLD can be intuitively evaluated, providing a reference for clinical diagnosis. *p* < 0.05 was considered significant statistically.

## 3 Results

### 3.1 Single factor analysis

**Figure 1:**
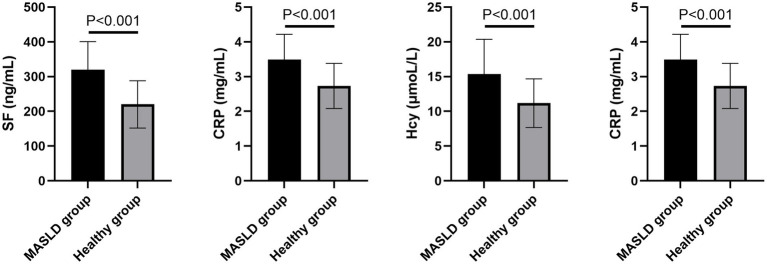
Comparison of serum SF, Hcy, CRP, and CAP levels between the two groups. An independent sample *t*-test was used for the comparison.

Single factor analysis showed that there were no significant differences exist in age, gender, smoking history, basic medical history, glucose, and lipid metabolism between the two groups (*p* > 0.05). However, the SF, Hcy, CRP, and CAP levels of the MASLD group were significantly higher than those of the healthy group (*p* < 0.05, Figure 1; Table 1).

**Table 1:** Single factor analysis between the two groups.

Factor		MASLD group (*n* = 102)	Healthy group (*n* = 102)	*χ*^2^/*t*	*p*
Gender [*n* (%)]	Male	88	85	0.342	0.558
	Female	14	17		
Age (year)	≤35	57	62	0.504	0.478
	>35	45	40		
BMI (kg/m^2^)	<28	76	79	0.242	0.623
	≥28	26	23		
Smoking history [*n* (%)]	yes	25	19	1.043	0.307
	no	77	83		
History of diabetes mellitus [*n* (%)]	yes	10	8	0.244	0.622
	no	92	94		
History of Cardiovascular Disease [*n* (%)]	yes	19	15	0.565	0.452
	no	83	87		
FBG (mmol/L)	≤7	97	100	1.331	0.445
	>7	5	2		
TG (mmol/L)	<1.7	56	60	0.320	0.572
	≥1.7	46	42		
TC (mmol/L)	<5.2	54	57	0.178	0.673
	≥5.2	48	45		
HDL-C (mmol/L)	<1.0	73	79	0.929	0.335
	≥1.0	29	23		
LDL-C (mmol/L)	≤3.37	51	56	0.491	0.483
	>3.37	51	46		
UA (μmol/L)	≤420/360	63	70	1.059	0.304
	>420/360	39	32		
Blood pressure (mmHg)	≤140/90	86	88	0.156	0.693
	>140/90	16	14		
BUN (mmol/L)	≤8.0/7.5	97	99	0.520	0.721
	>8.0/7.5	5	3		
CREA (μmol/L)	≤97/73	96	99	1.046	0.498
	>97/73	6	3		
GGT (U/L)	≤60/45	89	93	0.815	0.367
	>60/45	13	9		
SF (ng/mL)		320.30 ± 80.28	220.00 ± 68.30	9.611	<0.001
Hcy (μmoL/L)		15.36 ± 5.01	11.18 ± 3.50	6.905	<0.001
CRP (mg/mL)		3.49 ± 0.73	2.73 ± 0.65	7.833	<0.001
CAP (dB/m)		295.34 ± 78.32	224.22 ± 70.15	6.831	<0.001
ATT (dB/cm/MHz)		0.61 ± 0.20	0.56 ± 0.15	1.938	0.054

Independent sample *t*-test or *χ*^2^ test was used for comparison between the two groups.

### 3.2 Single factor screening

The indicators with *p* < 0.05 in the above table were screened for univariate analysis, and the results showed that SF, Hcy, CRP, and CAP levels may be the influencing factors of MASLD (*p* < 0.05, Table 2).

**Table 2:** Single factor screening.

Items	*B*	*S.E.*	*Wald*	*p*	OR	95% CI
SF	0.019	0.003	45.170	<0.001	1.019	1.014 ~ 1.025
Hcy	0.227	0.040	32.070	<0.001	1.255	1.160 ~ 1.357
CRP	1.620	0.263	37.909	<0.001	5.053	3.017 ~ 8.463
CAP	0.013	0.002	32.267	<0.001	1.013	1.008 ~ 1.017

### 3.3 Multivariate logistic regression analysis

The presence of MASLD was set as the dependent variable, and SF, Hcy, CRP, and CAP levels with statistical significance in the above single factor screening were set as independent variables, and their values were assigned. Multivariate Logistic analysis showed that SF, Hcy, CRP, and CAP levels were independent predictors of MASLD (*p* < 0.05, Tables 3, 4).

**Table 3:** Assignment.

Variables	Name	Assignment
Dependent variables	Group	1 = MASLD group, 0 = healthy group
Independent variables	SF	Measurement data were included according to actual values
	Hcy	Measurement data were included according to actual values
	CRP	Measurement data were included according to actual values
	CAP	Measurement data were included according to actual values

**Table 4:** Multivariate logistic regression analysis of the risk factors of MASLD.

Items	*B*	S.E.	Wald	*p*	OR	95% CI
SF	0.019	0.003	29.62	<0.001	1.019	1.012 ~ 1.026
Hcy	0.183	0.052	12.436	<0.001	1.201	1.085 ~ 1.330
CRP	1.717	0.390	19.424	<0.001	5.567	2.594 ~ 11.945
CAP	0.015	0.003	21.057	<0.001	1.015	1.009 ~ 1.022

### 3.4 The predictive value of serum SF, Hcy, CRP, CAP and the combination of indicators for MASLD

**Figure 2:**
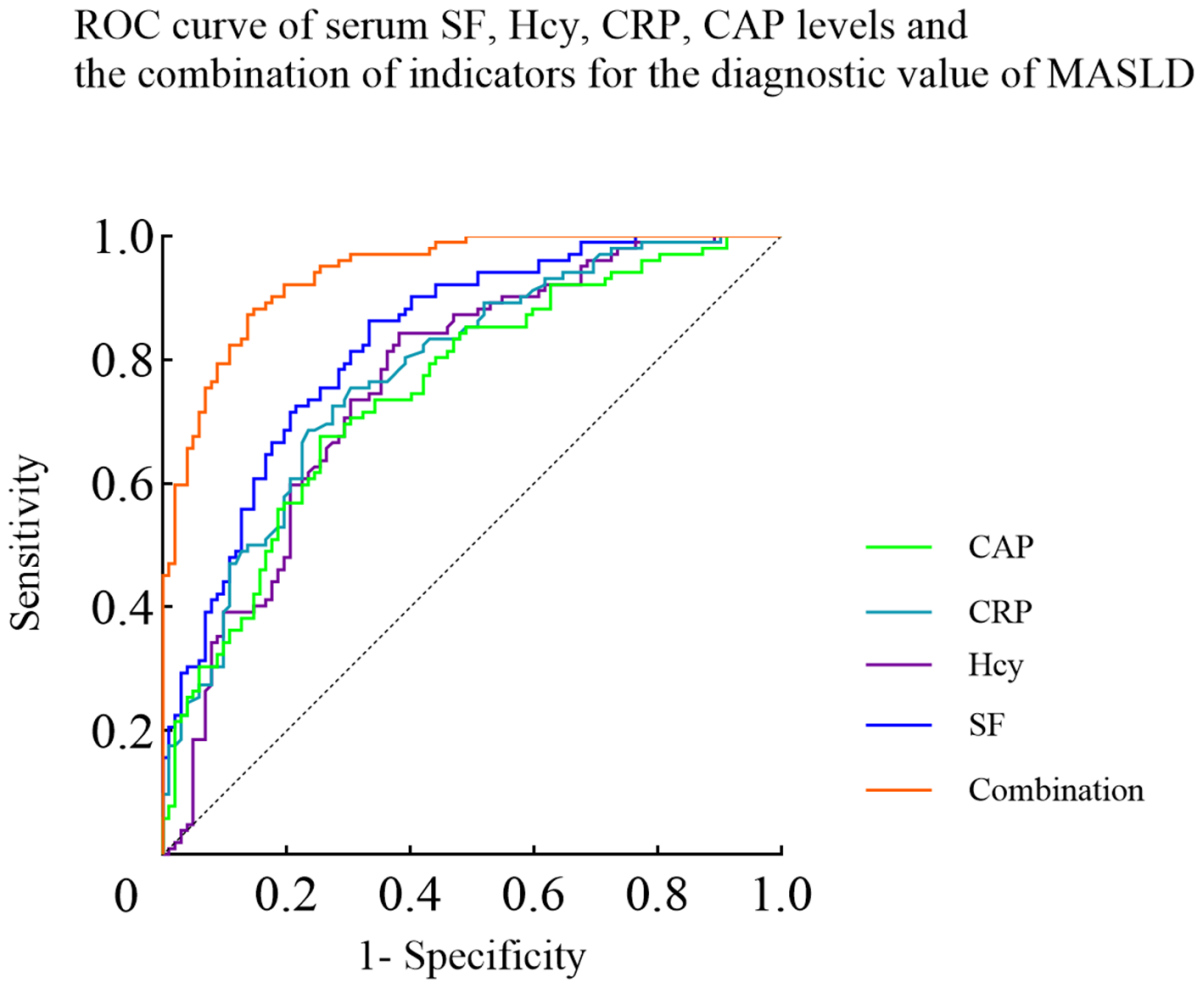
ROC curve of serum SF, Hcy, CRP, CAP levels and the combination of indicators for the diagnostic value of MASLD.

ROC curve displayed that the AUC of the combined prediction of SF, Hcy, CRP and CAP levels was 0.941, which was significantly higher than the values of obtained from individual predictions (0.829, 0.761, 0.780, 0.750). The sensitivities of the individual SF, Hcy, CRP, and CAP, as well as the sensitivity of the combined prediction, were 0.794, 0.716, 0.696, 0.745, and 0.892, respectively. The specificities of the individual SF, Hcy, CRP, and CAP, as well as the specificity of the combined prediction, were 0.716, 0.676, 0.755, 0.667, and 0.814, respectively (Table 5; Figure 2).

**Table 5:** Diagnostic value of serum SF, Hcy, CRP combined with fat ultrasound parameters and indexes for MASLD.

Variables	AUC	SE	*p*	95%*CI*	cut-off value	Youden index	Sensitivity	Specificity
SF	0.829	0.028	<0.001	0.774 ~ 0.884	257.865	0.510	0.794	0.716
Hcy	0.761	0.034	<0.001	0.695 ~ 0.827	12.585	0.392	0.716	0.676
CRP	0.780	0.032	<0.001	0.718 ~ 0.843	3.115	0.451	0.696	0.755
CAP	0.750	0.034	<0.001	0.684 ~ 0.817	246.505	0.412	0.745	0.667
Joint prediction	0.941	0.015	<0.001	0.912 ~ 0.970	0.395	0.706	0.892	0.814

## 4 Discussion

The liver, as an important metabolic hub in the human body, mainly responsible for the production and transportation of fat. When this metabolic balance is disturbed, fat will abnormally accumulate in the liver, thereby forming fatty liver. In the past, MASLD was considered as a relatively benign condition. However, in recent years, the increased risk of cardiovascular complications related to MASLD, kidney damage, and even the rising risk of cancer clearly indicate that it is no longer confined to the traditional benign category and is gradually attracting widespread attention from all walks of life. According to global statistics, 25% of the adult population suffer from MASLD, and the prevalence rate in Asia is as high as 27% (10, 11), and it is increasing year by year. It is particularly noteworthy that, with the growth of aging population and obesity, the prevalence of MASLD has increased significantly, and even the incidence rate in this group can reach 90%. Although liver biopsy is currently regarded as the “gold standard” for diagnosing MASLD, its invasive nature may lead to complications or adverse consequences. Therefore, in the diagnosis of MASLD, liver ultrasound has become a widely used diagnostic method due to its non-invasive nature, rapidity, and high patient acceptance (12).

The fat ultrasound parameter CAP has certain application value in the diagnosis of MASLD (13, 14), which can accurately detect and quantify the fat content in the liver and can be used to evaluate the degree of liver fatty lesions and fibrosis in MASLD patients (15). Mehnoosh et al. (13) divided MASLD patients into three groups and found that the greater the CAP value, the more severe the condition. The results of this study were similar to the above study, obtained by comparing the CAP values of MASLD group and healthy control group. The CAP value of the MASLD group was larger, but the ultrasound examination was also performed. In this study, there was no difference in the ATT value between the two groups, which may be due to the differences in detection methods and study samples. In addition, there may also be differences in the fat content or other components (such as water, fibrosis) in the tissue, which may lead to significant differences in CAP. The study by Eddowes et al. (16) showed that the AUC of CAP value reached 0.870, higher than the result obtained in this study (0.750). Even with high diagnostic value, the diagnostic results are often affected by the personal experience and interpretation ability of doctors, resulting in certain subjectivity and error space in image analysis (17). Therefore, it is necessary to combine ultrasound diagnosis with other indicators to predict the probability of MASLD, to improve the accuracy of diagnosis and provide reference for clinical diagnosis and treatment.

SF is the protein with the highest iron content in the human body and is often used for diagnosing anemia symptoms. The research conducted by Kowdley et al. (18) indicates that patients with higher levels of SF tend to affect the normal operation of liver function. The effect of SF on the liver mainly lies in its role as a regulator of iron metabolism. Under normal conditions, SF helps maintain the balance of iron in the body. If the content of SF is too high, it may lead to the deposition of iron in the liver and other organs, thereby causing liver cell damage, iron overload, and immune responses, and potentially leading to liver enlargement, liver fibrosis, and even cirrhosis. In this study, a control experiment was conducted by collecting medical records, and it was found that the serum SF concentration of the MASLD group was significantly higher than that of the healthy group, which was similar to the conclusion of George et al. (19). This may be because high levels of SF may affect insulin signaling or energy metabolic pathways, thereby exacerbating the difficulty in blood glucose regulation and promoting the development of metabolic syndrome (20). When the SF concentration is abnormally elevated, it not only promotes the development of MASLD, but also may indicate a disorder in iron metabolism. Hcy is an important intermediate product in the methionine metabolic process, and its main metabolic site is concentrated in the liver. The metabolism of Hcy involves two key pathways: one is through the process of reverse sulfation, converting methylated substances into methionine and then breaking it down into cysteine; the other depends on the expression of specific genes in the liver, including cysteine sulfohydrolase and cysteine *γ*-dehydrogenase, which are responsible for converting Hcy into cysteine (21). In addition, when the liver is damaged, the serum Hcy level will show significant changes, specifically manifested as an increase in Hcy concentration. This elevated Hcy level will further aggravate the liver damage process, forming a vicious cycle (22). The results of this study revealed that the concentration of Hcy in the MASLD group was significantly higher than that in the healthy group, and multivariate Logistic regression analysis indicated that high concentration of Hcy was a risk factor for MASLD, and had good discrimination. CRP is an acute phase protein, and its level usually increases in MASLD patients and is positively correlated with liver function indicators. The increase in CRP level may reflect the degree of liver inflammation and is an important marker for the progression of MASLD. Many studies have confirmed the association between high CRP and liver steatosis (23). For instance, in a case–control study on subclinical atherosclerosis and arterial stiffness in MASLD patients conducted by Tahabet S et al. in the Journal of Vascular Medicine in May 2021 (24), it was pointed out that the CRP level of MASLD patients was significantly increased, and this increase was closely related to the degree of liver steatosis. Through a case–control analysis of the Algerian population, this study further confirmed the important role of C-reactive protein (CRP) as an inflammatory marker in reflecting the inflammatory process related to liver steatosis, which is consistent with our research results on the changes in CRP levels in MASLD patients. The study by Calabro et al. (25) showed that the CRP concentration in patients with fatty liver was significantly higher than that in the healthy control group, which was similar to the results of this study. This is because that patients with fatty liver may have excessive fat accumulation in liver cells, which may lead to liver cell damage and trigger an inflammatory response. As an important marker of the inflammatory response, the CRP level in patients with fatty liver may increase, reflecting the inflammatory state of the liver (26). However, in the study of Calabro et al., the sensitivity of this index was 0.800, higher than the results of this study, and the specificity was 0.400, lower than the results of this study. The reason for this may be that the diagnostic criteria or gold standard used in the current study are different from those in previous studies, resulting in the difference. ROC curve analysis showed that the AUC value of the combined prediction of SF, Hcy, CRP, and CAP was 0.941, indicating that the combination of these indicators has good predictive value.

In the era of rapid development of genomics and advanced imaging technologies, research on MASLD has been continuously deepened. Many biomarkers have been proposed for the diagnosis and assessment of the disease. However, most current studies focus on a single biomarker or a single detection technology, which has certain limitations in comprehensively and accurately reflecting the complex pathological and physiological process of MASLD. This study employed a comprehensive biomarker approach, integrating serological indicators (SF, Hcy, CRP) with fat ultrasound parameters (CAP), to evaluate MASLD from various perspectives. Serological indicators can reflect physiological and pathological changes such as inflammatory responses and metabolic disorders within the body, while CAP directly reflects the changes in liver fat content. This multi-dimensional and comprehensive assessment method is closer to the actual pathogenesis of MASLD and provides a new perspective for in-depth understanding of the essence of this disease. Through this study, we further confirmed the effectiveness of the comprehensive biomarker method in the diagnosis of MASLD, enriched the diagnostic strategies in this field, and promoted the transition from single indicator-based diagnosis to multi-indicator comprehensive assessment for MASLD.

There are currently numerous studies on biomarkers for MASLD. Although each of these studies can to some extent reflect certain characteristics of the disease, a single biomarker is often affected by multiple factors, resulting in limited specificity and sensitivity for diagnosis. The comprehensive biomarker method in this study effectively leverages the advantages of each indicator by integrating serumological indicators and fat ultrasound parameters, achieving mutual supplementation. SF, Hcy, CRP, respectively, reflect the pathological state of the body from aspects such as iron metabolism, amino acid metabolism, and inflammatory response, while CAP directly shows the liver fat deposition situation. The results of multivariate logistic regression analysis and ROC curves indicate that the area under the curve of the combined prediction (0.941) is significantly higher than that of each indicator alone, suggesting that the comprehensive biomarker method can more comprehensively and accurately capture the characteristics of MASLD, significantly improving the diagnostic accuracy and reducing the occurrence of misdiagnosis and missed diagnosis.

In clinical practice, simple, cost-effective and efficient diagnostic methods are of vital importance for the early screening and intervention of diseases. Some of the existing advanced biomarker detection methods, such as those based on genomics or proteomics, although having high sensitivity and specificity, often require complex experimental equipment, professional technicians and high detection costs, which limits their wide application in grassroots medical institutions. The serological index tests and fat ultrasound examinations adopted in this study are all routine clinical examination items, featuring simple operation, low cost, and strong repeatability. The serological index tests can be completed through routine blood collection and biochemical analysis, while the fat ultrasound examination utilizes common ultrasound equipment and does not require special instruments or techniques. This comprehensive biomarker method is not only applicable to large medical institutions but can also be promoted and applied in primary hospitals and community health service centers, providing a more convenient and economical means for the early diagnosis and intervention of MASLD, and enhancing its applicability in clinical practice.

In conclusion, the combined application of serum SF, Hcy, CRP, and CAP has a high predictive efficiency for MASLD and can be used as an important reference indicator for early clinical diagnosis and intervention. Future research can further explore the changes of these indicators in different pathological stages of MASLD and their interaction mechanisms to optimize the diagnostic strategy and improve the prognosis of patients. However, this study still has many shortcomings. The sample size is limited, which may affect the wide applicability of the results. The lack of longitudinal data makes it impossible to observe the changes of the indicators dynamically; the interaction of multiple factors on MASLD has not been fully explored. Moreover, there are differences between the diagnostic criteria and the gold standard, which may lead to bias. In the future, the sample size should be expanded and multi-center cooperation should be carried out. Longitudinal studies have been conducted to observe the changes of the indicators dynamically. Further exploration of the interaction of multiple factors is needed to deepen the understanding of the pathogenesis of MASLD. The use of new technologies to improve the diagnostic accuracy of MASLD can achieve early diagnosis and precise treatment of MASLD, thereby improving the prognosis. These efforts will help to improve the scientificity and effectiveness of the MASLD network.

## Data Availability

The datasets presented in this study can be found in online repositories. The names of the repository/repositories and accession number(s) can be found in the article/supplementary material.
